# Tissue degeneration in ALS affected spinal cord evaluated by Raman spectroscopy

**DOI:** 10.1038/s41598-018-31469-4

**Published:** 2018-08-30

**Authors:** Gennaro Picardi, Alida Spalloni, Amanda Generosi, Barbara Paci, Nicola Biagio Mercuri, Marco Luce, Patrizia Longone, Antonio Cricenti

**Affiliations:** 1 CNR Istituto Struttura della Materia, Via Fosso del Cavaliere 100, I-00133 Rome, Italy; 2 Laboratorio di Neurobiologia Molecolare, Fondazione Santa Lucia IRCCS, Via del Fosso di Fiorano 64/65, I-00143 Rome, Italy; 3 Department of Systems Medicine, Neurology UOC, University of Rome “Tor Vergata”, Fondazione PTV, Policlinico“Tor Vergata”, Viale Oxford 81, I-00133 Rome, Italy; 4 Department of Experimental Neuroscience, Fondazione Santa Lucia IRCCS, Via del Fosso di Fiorano 64/65, I-00143 Rome, Italy

## Abstract

The Raman spectral features from spinal cord tissue sections of transgenic, ALS model mice and non-transgenic mice were compared using 457 nm excitation line, profiting from the favourable signal intensity obtained in the molecular fingerprint region at this wavelength. Transverse sections from four SOD1G93A mice at 75 days and from two at 90 days after birth were analysed and compared with sections of similarly aged control mice. The spectra acquired within the grey matter of tissue sections from the diseased mice is markedly different from the grey matter signature of healthy mice. In particular, we observe an intensity increase in the spectral windows 450–650 cm^−1^ and 1050–1200 cm^−1^, accompanied by an intensity decrease in the lipid contributions at ~1660 cm^−1^, ~1440 cm^−1^ and ~1300 cm^−1^. Axons demyelination, loss of lipid structural order and the proliferation and aggregation of branched proteoglycans are related to the observed spectral modifications. Furthermore, the grey and white matter components of the spinal cord sections could also be spectrally distinguished, based on the relative intensity of characteristic lipid and protein bands. Raman spectra acquired from the white matter regions of the SOD1G93A mice closely resembles those from control mice.

## Introduction

Amyotrophic lateral sclerosis (ALS) is a very common degenerative disease of the motor neuron system, affecting it at different levels. Lower motor neurons, residing in the ventral horns of the spinal cord and in the brain stem are affected, as well as corticospinal upper motor neurons, residing in the precentral gyrus. Frequently, prefrontal motor neurons, involved in coordinating the functions of upper and lower motor neurons are also concerned^1–3^. Typically, initial symptoms are the dysfunction or weakness in one part of the body, following which the disease spreads to other parts of the body. The progressive loss of motor neurons and the dismantling of the motor neuron system is incurable and fatal due to respiratory failure, with a median survival of 3 years^4^. The only two drugs approved for the disease are riluzole and edaravone, both with mild to little effects on survival and quality of life^5,6^. To assess the presence of upper and/or lower motor neuron or cranial nuclei dysfunction, a combination of laboratory, radiographic, and even genetic background data are used, including electromyography, pulmonary function tests, motor speech evaluations, and neurologic exam to reveals potential cognitive and behavioural impact of symptoms in ALS. Brain and spinal cord Magnetic Resonance imaging (MRI) analysis is also used to assess whether atrophy in those regions, a characteristic of ALS, is present. Still, analytical tools are highly demanded for an earlier diagnosis and to evaluate the impact of therapeutics on the disease progression.

Motor neurons are located in the ventral horns of the spinal cord, which is composed of white and grey matter. In a transverse cross section of the spinal cord, the grey matter is shaped as a butterfly (or H), with the white matter filling the rest of the circular section. Different regions of the spinal cord are further classified: the dorsal horns and the ventral horns form the wings of the butterfly, connected by a central, internal column. The white matter is divided in dorsal, lateral and ventral columns; it is composed mostly of myelinated axons transmitting information by electrical pulses. The electrically insulating myelin sheet is very rich in lipids and is responsible for the white colour. The composition of the grey matter is more varied, consisting also of various type of endothelial, nerve and (more numerous) glial cells having different support functions and blood vessels.

Raman Spectroscopy is a valuable technique for biomedical applications^7–9^. The biochemical composition of tissue samples can be retrieved through the analysis of the characteristic molecular fingerprints contributing to the Raman spectrum. Modifications in the spatial distribution or in the relative ratio of components (e.g., lipids, proteins, nucleic acids) are adopted as benchmark for discriminating between normal and pathological tissue and/or for monitoring disease progression at molecular level. Many tissue types have been studied by Raman spectroscopy, but there are rather few reports on spinal cord tissue and only in connection with pathological alterations due to spinal cord injury (SCI) following blunt trauma or laceration. Saxena *et al*. first used Raman spectroscopy (785 nm excitation) to study biochemical changes due to SCI in a rat model^10^. The observed spectral differences between healthy and injured spinal cord were associated to axons demyelination and upregulation of chondroitin sulfate proteoglycans (PG) in the extracellular matrix at the site of the injury. Combining Fourier Transform Infrared Spectroscopy, spontaneous Raman spectroscopy and non-linear Coherent Anti Stokes Raman (CARS) imaging, Galli *et al*. identified normal and altered regions in a rat model of SCI^11^. The lesion and surrounding tissue were characterized by a decrease in intensity of the vibrational bands related to lipids, accounting for demyelination. Wang *et al*. evidenced by confocal Raman imaging the variations of lipid content in grey and white matter and the higher degree of vascularization in grey matter in healthy human tissue using 633 nm excitation^12,13^. Specifically concerning ALS, a recent study appeared in which Stimulated Raman Scattering (SRS) was used to image the sciatic nerve (connected by ganglions to the spinal cord) in transgenic mouse models carrying the SOD1G93A gene associated with human ALS^14^. SRS (as CARS) is mostly sensitive to lipid vibrations and is employed to image lipid distributions in biological samples. The SRS images associated an accumulation of lipid ovoids, most likely derived from degenerating myelinating cells, with early nerve degeneration.

Here, high quality confocal Raman spectra from spinal cord tissue sections of mouse models were recorded for the first time using 457 nm laser excitation. Normally, lowering the wavelength of the laser source increases the cross section of the Raman process, as this is proportional to the inverse fourth power of the used wavelength. Fluorescence contribution may be lessened with extended laser exposure. This is achieved with minimal to no sample damage with concentrated power densities. The spectra from control, healthy mice and transgenic SOD1G93A mice were compared to assess the potential of Raman spectroscopy with 457 nm excitation for the early detection of the degenerative disease.

## Results

### Control samples

**Figure 1:**
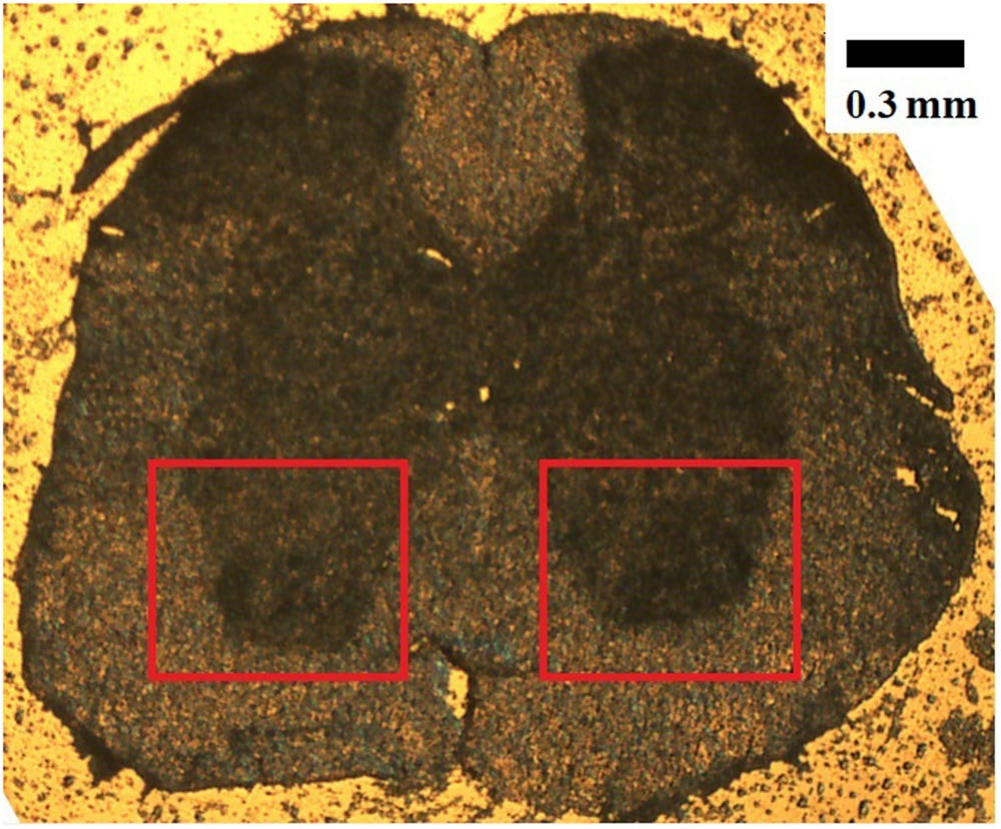
Representative microphotograph (5X objective) of a mouse spinal cord tissue section in the lumbar tract (L1-L2). The dorsal half section is up; the ventral half is down. The scale bar is 0.3 mm. The red boxes indicate the area of the ventral horn (where the cell bodies of the motor neurons reside) that were probed.

**Figure 2:**
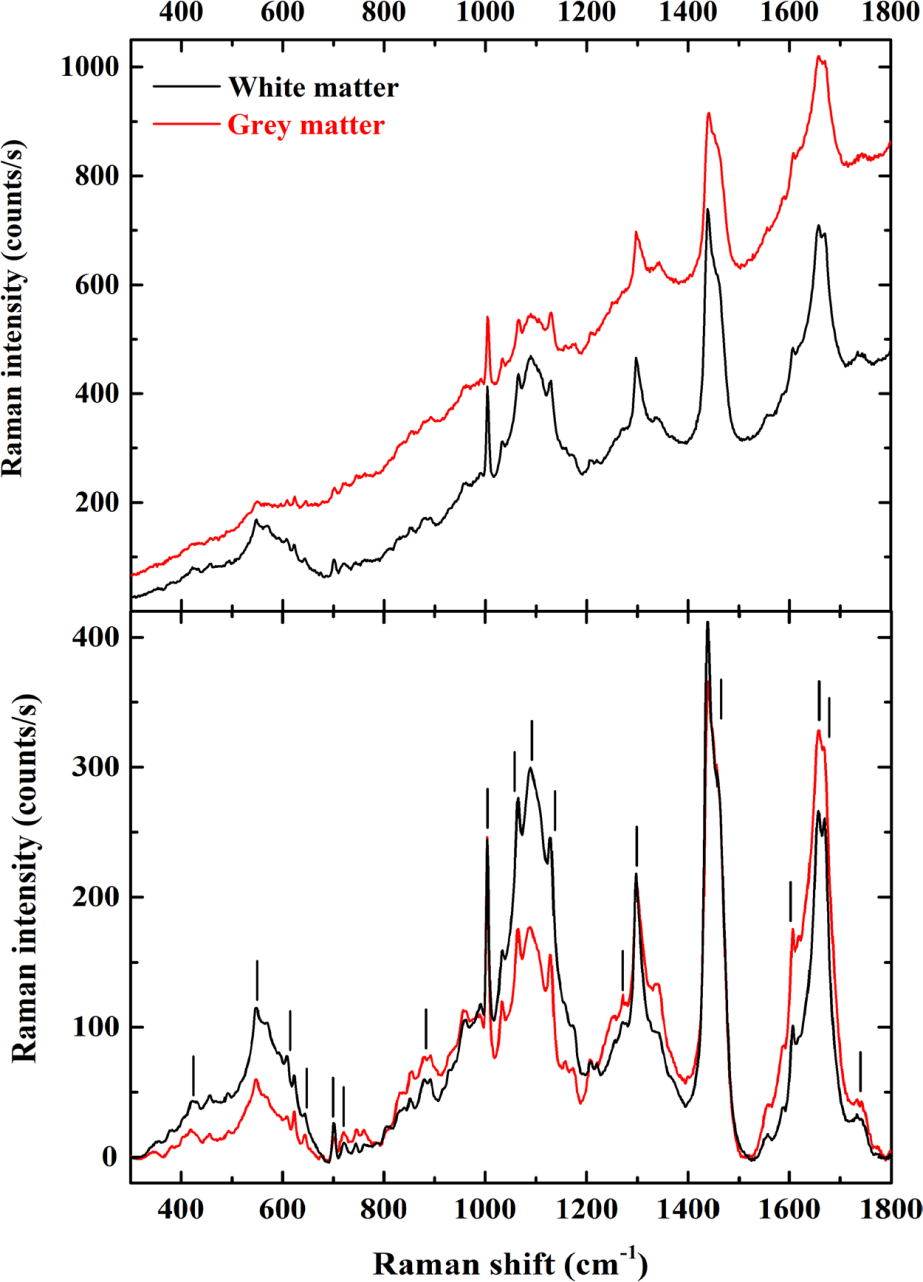
(**above**) Representative Raman spectra from the white matter region (black trace) and from the grey matter (red trace) of healthy mouse spinal cord section. The exposure time was 10 seconds for 12 accumulations with 6 mW/μm^2^ laser power. (**below**) Baseline corrected trace obtained averaging 21 white matter spectra (in black) and 21 grey matter spectra (in red) of healthy mouse spinal cord section. The marked bands are identified in the supplementary information.

A microscope image of a spinal cord section from a healthy mouse is presented in Fig. 1, showing the central, butterfly shaped grey matter area, surrounded by white matter. The red boxes in Fig. 1 indicate the areas of gray matter that have been probed, comprising the ventral horns containing the motor neurons cell bodies. This is the area which amply degenerate in ALS. The white matter investigated was directly surrounding the ventral horns, comprising the lateral and ventral columns. The Raman spectral features from healthy mice, used as reference, are discussed first. The spectra were highly reproducible within the white matter regions (less than 20% variations in the overall intensity of the baseline corrected signal), with different areas (lateral and ventral) providing nearly identical traces. Furthermore, the intensity in the fingerprints region was ~400 counts/s compared to ~20 counts/sec detected using 785 nm excitation for comparable laser power (supplementary information), enabling the acquisition of very detailed spectra. The black trace in Fig. 2(*above*) is a representative Raman spectrum from the white matter, before baseline correction. The black trace in Fig. 2(*below*) is the average between 26 spectra acquired from different white matter spots on tissue sections of five different control mice. Each individual spectrum was baseline corrected with a 3^rd^ order polynomial curve. The general spectral profiles are similar to those presented by Saxena *et al*.^10^ at 785 nm and Wang *et al*. at 633 nm^12^, although in our case several additional details were observed thanks to the more favourable signal-to-noise ratio. Table 1 in the supplementary material reports a list of the major Raman frequencies observed, with assignment.

The bands related to lipids are the most intense and readily identifiable: the methylene (-CH_2_-) twisting and wagging at 1298 cm^−1^, the methylene scissoring at 1439 cm^−1^ with a shoulder at 1458 cm^−1^ due to the methyl (-CH_3_) asymmetric bending modes and the skeletal stretching modes at 1127 cm^−1^, 1064 cm^−1^ and at ~1089 cm^−1^. This last broader feature also includes the phosphate stretching vibrations in the phospholipids. Other relevant but weak features that can be assigned to phospholipids or cholesterol are at 877 cm^−1^, 719 cm^−1^, 701 cm^−1^, 548 cm^−1^ and 424 cm^−1^ ^15,16^. The sharpest feature at 1003 cm^−1^ is the benzene ring breathing of phenylalanine, to which a weaker but equally sharp feature at 1606 cm^−1^ is associated. The intense doublet at 1668 cm^−1^ and 1656 cm^−1^ are the amide I bands in *β*-sheets and *α*-helix (respectively) of proteins, though lipids may also contribute to these bands^17^. The much weaker amide III for *α*-helix is detected at 1271 cm^−1^ and possibly at 1251 cm^−1^ for unordered structures.

Figure 2 (red traces) also displays Raman spectra of the grey matter from the ventral horns region. There are substantial variations in the relative bands intensity between white and grey matter, previously not fully recognized. The amide I doublet near 1660 cm^−1^, convolving contributions from various protein structures, gained in intensity in the grey matter spectrum; while the broad, complex structures between 510 cm^−1^ and 670 cm^−1^ and between 1020 cm^−1^ and 1150 cm^−1^ (both mostly related to various lipid contributions) are more intense in the white matter spectrum. These first observations are indicative of the different biochemical environment: on one hand, a higher lipids content in the white matter and, on the other hand, the presence of protein rich nerve and glial cells in the grey matter. There are further, subtler differences, which can be used to differentiate individual spectra from either the white and or the grey matter. Within the lipid related spectral feature located between 1420 cm^−1^ and 1500 cm^−1^, the sharp component at 1438 cm^−1^ is more prominent in the white matter than in the grey matter. This is also evident in the spectra reported by Galli *et al*. using 785 nm excitation^11^. Apart from the absolute intensity, the profile of the amide I band slightly changes, indicating that distinct secondary structures are present in different proportions in the white and in the grey matter. Despite identical bands are seen in the Raman signals of the white and grey matter, their respective spectra are distinguishable based on the mentioned intensity disparities.

### Analyses of SOD1G93A spinal cord sections

Next, Raman spectra were acquired from spinal cord sections of SOD1G93A mouse models. The time points analysed in the study are postnatal days 75 (P75) and postnatal days 90 (P90). Behaviourally, at P75 mice are still able to mate and perform on the rotarod as a control; their weight is not statistically dissimilar from the control mice. In general the transgenes do not present any visible phenotypic signs of the disease. A total of seven sections from the spinal cords of four mice at P75 were probed, and compared to the sections from control mice of the same age. On each section, 8–10 spectra were acquired along a line running through a ventral horn (from the ventral white region to a lateral white region), the separation between two consecutive locations was ~50 μm.

**Figure 3:**
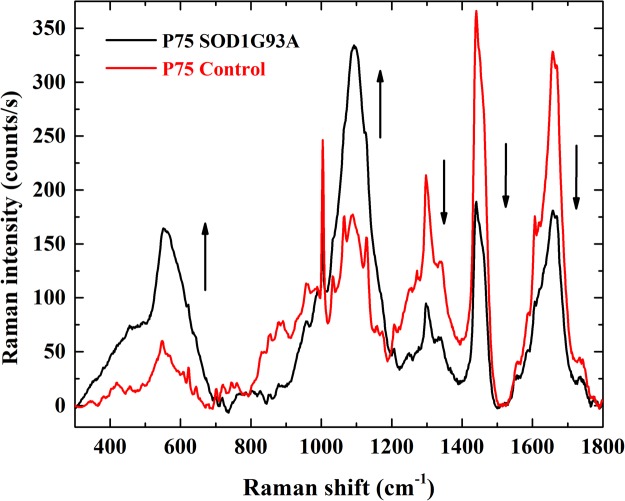
Baseline corrected Raman spectra from the grey matter region within the ventral horns of P75 healthy mouse spinal cord section (red trace) and of a P75 SOD1 mouse models (black trace).

**Figure 4:**
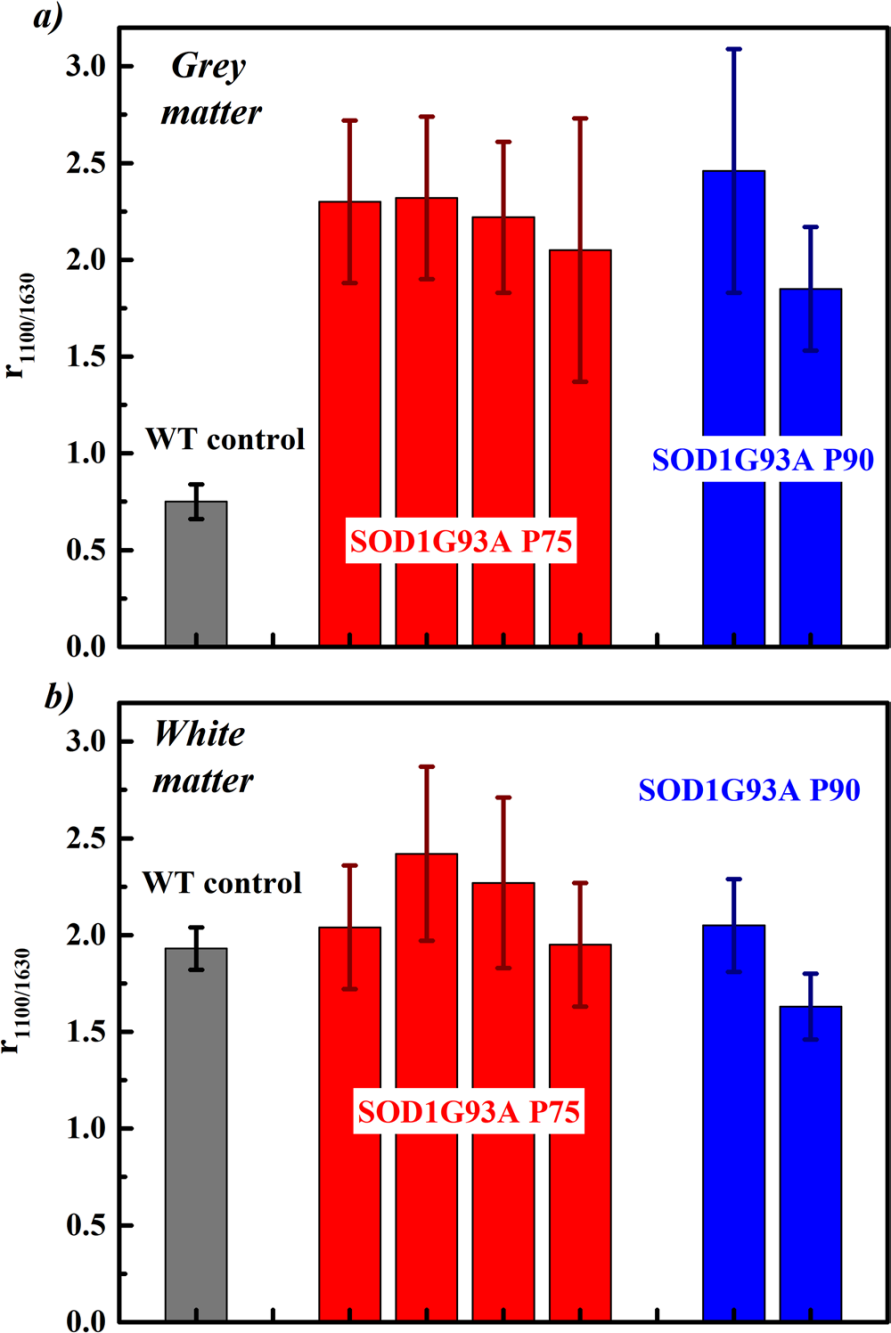
Histogram reporting the Raman signal intensity ratio between the spectral envelopes centred at 1100 cm^−1^ and at 1630 cm^−1^ for the analysed spinal cord sections of WT control mice and of SODG93A P75 and P90 models. The mean value and standard deviation reported for the WT control were obtained considering all the spectra recorded from the spinal cord sections of five mice. For the SOD1G93A models, each column is relative to a different specimen. Panel *(a)* is relative to grey matter regions in the ventral horns. Panel *(b)* is for white matter regions of the same spinal cord sections as in *(a)*.

Marked differences are evident when comparing with spectra from healthy tissue. Figure 3 reports the spectra from the grey matter region, ventral horns, of healthy mouse (red trace) and of a P75 SOD1G93A mouse model (black trace, average of 17 spectra, from three different sections of the same spinal cord). With respect to the healthy section, there is an overall intensity increase in the spectral window from 300 cm^−1^ to 700 cm^−1^ and between 1050 cm^−1^ and 1200 cm^−1^. On the opposite, the other three main groups of bands with central position at ~1300 cm^−1^, ~1450 cm^−1^ and ~1630 cm^−1^ are much less intense in the spectrum from the diseased section. For a first quantitative estimation of the spectral modification within the grey matter, the ratio *r*_1100/1630_ between the integral intensities of the band envelope centred at ~1100 cm^−1^, CC skeletal modes of lipids, proteins and the envelope at ~1630 cm^−1^, amide bands of proteins is considered. A value of 0.75 ± 0.09 (standard deviation over five mice) is for the healthy mice, while a value of 2.3 ± 0.4 is for the black trace of Fig. 3, relative to the diseased mouse at P75. The SOD1G93A model has been engineered to develop ALS in a definite time frame and with specific characteristics resembling human ALS^18^. Yet, it is feasible to imagine that between separate individuals differences in the degree of penetrance of the mutant human SOD1G93A gene and other features can affect the spinal cord tissue of different mouse to various extent, regardless of identical age and growth conditions^19^. Thus, the biomolecular composition of the grey matter in the spinal cord of diseased mice shows larger variability between individuals, with respect to the grey matter of healthy mice. The ratio *r*_1100/1630_ was 2.3 ± 0.4, 2.2 ± 0.4 and 2.01 ± 0.7 for the tissue sections from the other three P75 transgenic mice (Fig. 4a).

Furthermore, we notice a loss in the sharpness of the individual spectral features: several distinct peaks previously discernible are now merged in broader envelopes (especially below 1200 cm^−1^, contributions from the skeletal modes in lipids). The increase in the bandwidths can be interpreted as being related to a structural relaxation or increased molecular disorder. A lower degree of order in the orientation of the myelinated nerve fibre and a decreased lipid packing are further signals of the on-going tissue degeneration^20,21^.

The increased Raman intensity between 400 cm^−1^ and 690 cm^−1^ is possibly related to the proliferation of proteoglycans in the grey matter. Previously, in their Raman investigation of the spinal cord tissue from rat models affected by SCI, Saxena *et al*. highlighted an increased peak area for two bands roughly centred at 510 cm^−1^ and 615 cm^−1^ and assigned to aggregated proteoglycans (PG) and Glycosaminoglycans (GAG)^10,22^. This was based on a previous Raman study on aggrecan (a PG with mostly chondroitin sulphate side chains) in monomeric and aggregagated form, individuating in particular three bands at 642 cm^−1^ (tyrosine), at 582 cm^−1^ (PO deformation) and at 510 cm^−1^ (unassigned). Similarly, PG vibrations may contribute to the increased intensity of the envelope centred at 1100 cm^−1^, in particular a component at ~1065 cm^−1^ ^22^. Proteoglycans bearing GAG side chains are secreted by invasive astrocytes, eventually leading to gliosis and the formation of a glial scar within the injured spinal cord. Together with axons demyelination, formation of the glial scar is the other hallmark of SCI. In ALS, collagen abnormalities of skin have been reported among patients. A study by Ono *et al*. on the morphological and biochemical characteristics of skin from 8 ALS patients found an increased amount of hyaluronic acid and a significant positive correlation between its content and the duration of the illness^23^. They concluded that GAG metabolism could be altered in the skin of patients with ALS. Moreover, chondroitin sulfate proteoglycans (CSPGs), major components of the extracellular matrix in the central nervous system, are up-regulated in the spinal cord of transgenic of transgenic rats with His46Arg mutation in the Cu/Zn superoxide dismutase (SOD) gene, a rat model of ALS^24^. The accumulation of CSPGs is probably participated by reactive astrocytes, a loop that in turn may contribute to create a hostile microenvironment for neural survival and regeneration in ALS. They are one of the most abundant glycanated protein types found in the nervous system with an inhibitory role in neural plasticity due to their formation in perineuronal nets, dense lattice-like structures^25^. Although the up-regulation of CSPGs following an insult or in neurodegenerative processes is thought to be a protective mechanism, an attempt to segregate the damage and limit its spread^26^, it actually generates an environment hostile to regeneration and repair. Hence the increase in proteoglycans that we have observed could be seen as a sign of the progressive neurodegenerative processes showing reactive astrogliosis and the deposition of CSPGs. It is also important to consider that in response to a central nervous system damage, either by trauma or disease processes, proinflammatory cytokines released by activated glial cells, stimulate the upregulation of CSPGs expression, helping the formation of the glial scar^27^. Initially, this could be seen as a protective response that turns detrimental since it inhibits axonal sprouting, regeneration and synaptic transmission. Hence, the development of therapies aiming at controlling CSPGs formation may be considered important in helping to restore proper neuronal function in neurodegeneration.

For comparison purposes, within the white matter regions the spectral ratio amounts to 1.9 ± 0.1 for healthy mice. The spectra acquired from the white matter region of the SOD1G93A models resemble more closely to the ones from the controls, indicating less alterations in these regions. Still using the ratio *r*_1100/1630_ as a measure of the relative changes in the bands intensity, this increases only moderately (Fig. 4b). Sections from the lumbar tract of the spinal cord of P90 SOD1G93A mice display an analogous increase in the *r*_1100/1630_ ratio within the grey matter as for P75 samples, indicating that the compositional and structural changes detected by Raman spectroscopy have mostly developed within the first 75 days.

In conclusion, intense Raman signal in the molecular fingerprint region and detailed spectra were obtained from mice spinal cord tissue sections employing 457 nm excitation line. Spectra from the grey matter ventral horns of transgenic SOD1G93A, ALS model mice conspicuously differ from the spectra acquired from control, non-transgenic mice. At a biomolecular level, the observed spectral modifications were tentatively associated to the degradation in lipid structural order, the axons demyelination and the accumulation of intrusive proteoglycans, signalling the progression of the neurodegenerative disease. The time points analysed in the study, 75 and 90 postnatal days are particularly significant since, with respect to this model, >90 days are usually necessary to phenotypically diagnose ALS related symptoms. Based on these results, the adoption of the Raman signal relative intensity in the spectral windows 450–650 cm^−1^ and 1050–1200 cm^−1^ as ALS markers in the spinal cord is suggested for an earlier disease detection.

## Methods

### Animals and tissue preparation

The mouse strain used in the study is the B6SJL-TgN(SOD1-G93A)1Gur mice expressing the human G93A Cu/Zn superoxide dismutase (SOD) mutation originally obtained from the Jackson Laboratories (Bar Harbor, ME, USA)^18^. Selective breeding, at the Saint Lucia Foundation animal facility, maintained the transgene in the C57BL/6 J background (for >10 generations); controls were the nontransgenic C57BL/6 J wild type (WT) littermates. The Fondazione Santa Lucia Committee on Animal Research and Animal Care approved the protocols and procedures used in this study, in accordance with the European Union directive 2010/63/EU and the Italian Animal Welfare Act for the use and care of laboratory animals. The presence of the human transgene was performed as described in Spalloni *et al*.^28^. Following anesthetization with chloral hydrate, (400 mg/kg) adult (75, 90 postnatal days) WT and SOD1G93A mice were perfused intracardially with ice-cold 0.9% NaCl followed by 4% paraformaldehyde (PAF) in phosphate buffer (PB, 0.1 M, pH 7.2). The dissected vertebral columns were left in ice-cold PAF for 16 h, then immersed in filtered 30% sucrose PB for cryoprotection. The analyses have been performed on selected transverse sections (~40 μm in thickness) of the lumbar tract (L1-L2) of the spinal cord^29^.

### Raman microscopy

The Raman spectra were acquired on an InVia spectrometer from Renishaw LTD equipped with a solid state laser source emitting at 457.9 nm. A 50 X, 0.48 NA long working distance microscope objective was used for illumination and collection in back scattering configuration. Acquisition time was set at 10 s with 12 accumulations per spectrum. Laser density at the focus was ~6 mW/μm^2^. Each spot was pre-photobleached by illuminating for 6–8 minutes at this power density prior to each spectral acquisition, to lower fluorescence background contribution. Fluorescence in the green/yellow range is mostly due to the aldehyde fixative. The Raman features become more evident as the background decreases. After 8 minutes, the baseline and Raman spectral features display stable intensity.

## Electronic supplementary material

Supplementary Information

## Data Availability

The datasets generated and analysed during the current study are available from the corresponding author on reasonable request.
