# Supplementary material for: Tissue degeneration in ALS affected spinal cord evaluated by Raman spectroscopy
Source: Sci Rep. 2018 Aug 30;8:13110. doi: 10.1038/s41598-018-31469-4 (PMC6117324; doi:10.1038/s41598-018-31469-4)
Supplement: Supplementary file 1 — Supplementary Information [file 41598_2018_31469_MOESM1_ESM.docx]

Supplementary information

**Tissue degeneration in ALS affected spinal cord evaluated by**

**Raman spectroscopy**

Gennaro Picardi^1^, Alida Spalloni^2^,

Amanda Generosi^1^, Barbara Paci^1^, Nicola Biagio Mercuri^3,4^, Marco Luce^1^, Patrizia Longone*^2^*, Antonio Cricenti^1^

*^1^ CNR Istituto Struttura della Materia, Via Fosso del Cavaliere 100, I-00133 Rome, Italy*

*^2^* *Laboratorio di Neurobiologia Molecolare. Fondazione Santa Lucia.*

*Via del Fosso di Fiorano 64/65. I-00143 Rome, Italy*

*^3^Department of Systems Medicine, Neurology UOC, University of Rome “Tor Vergata”, Fondazione PTV, Policlinico“Tor Vergata”, Viale Oxford 81, I-00133 Rome, Italy*

*^4^Department of Experimental Neuroscience, Fondazione Santa Lucia,*

*Via del Fosso di Fiorano 64/65, I-00143 Rome Italy.*

Vibrational Frequencies in cm^-1^ and tentative assignment of selected Raman bands from spinal cord tissue section.

| Peak Position  (cm^-1^) | Assignement |
| --- | --- |
| 1741 | -CO_2_H, -CO_2_R |
| 1668 | ν(C=O) of amide I (*β*-sheet) in proteins or ν(C=C) in cholesterol ester |
| 1656 | ν(C=O) of amide I (*α*-helix) in proteins or ν(C=C) in lipids |
| 1606 | ν(C=C) in Phenylalanine |
| 1458 | CH_3_ bending in lipids |
| 1438 | CH_2_ scissoring in lipids |
| 1298 | CH_2_ twisting, wagging in lipids |
| 1272 | Amide III (*α*-helix) in proteins |
| 1127 | ν(C-C) in lipids |
| 1089-1104 | ν(C-C) in lipids + ν(P-O) |
| 1064 | ν(C-C) in lipids |
| 1003 | ν(C-C) in Phenylalanine |
| 877 | ν_as_(^+^N(CH_3_)_3_) in phosphatyildycholine |
| 719 | ν_s_(^+^N(CH_3_)_3_) in phosphatyildycholine |
| 701 | Ring deformation in cholesterol |
| 643 | Tyrosine |
| 622 | Polysaccharides (GAG) |
| 548 | Ring bending in cholesterol |
| 424 | Ring bending in cholesterol |


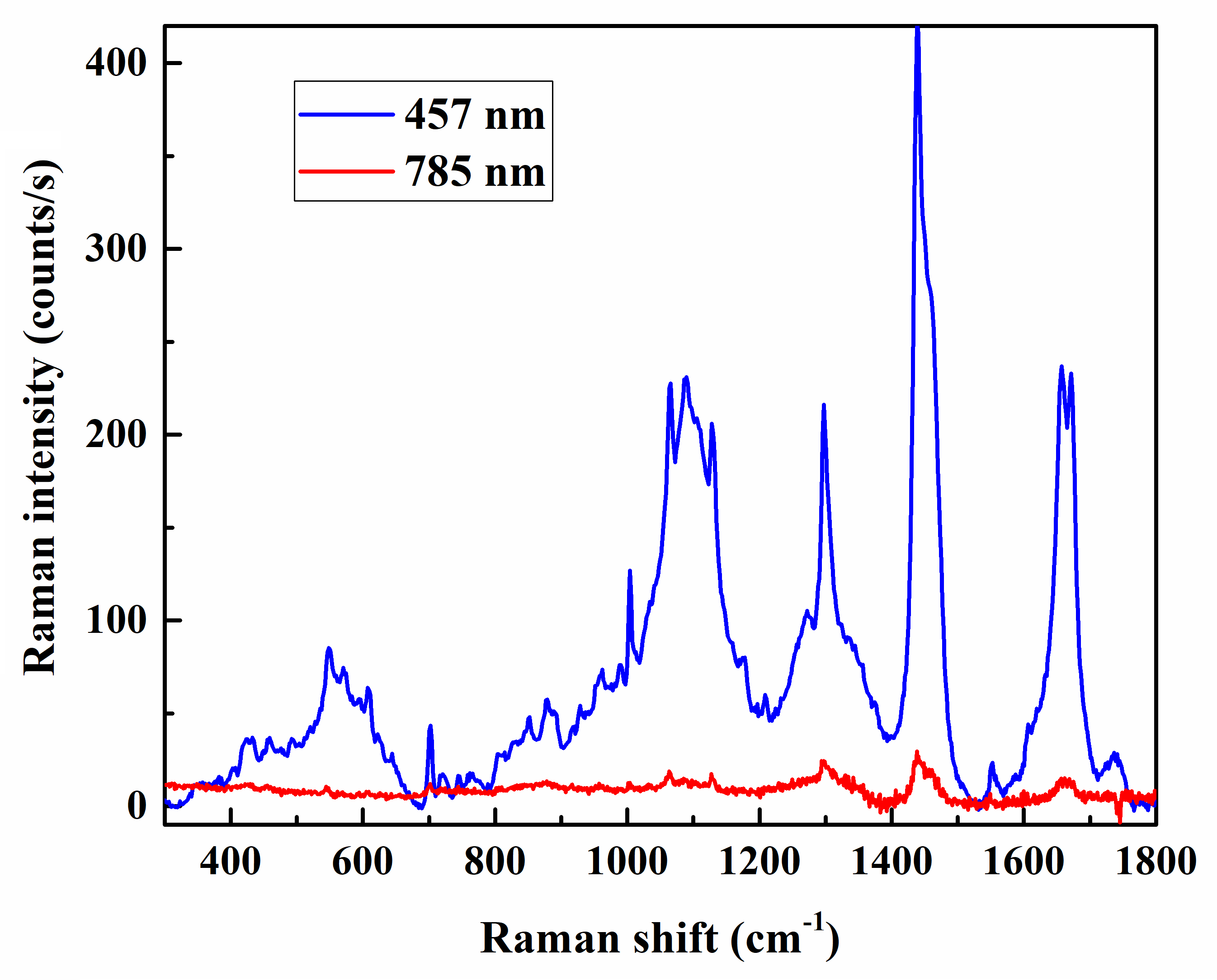


Baseline corrected Raman spectra from the white matter region recorded with 457 nm and 785 nm excitation lines.
